# First year experiences with a palliative out-patients structure for patients with COPD: a qualitative study of health professionals’ expectations and experiences

**DOI:** 10.1186/s12904-018-0369-2

**Published:** 2018-10-08

**Authors:** Dorthe Gaby Bove, Marie Lavesen, Maria Omel Jellington, Kristoffer Bastrup-Madsen Marsaa, Suzanne Forsyth Herling

**Affiliations:** 1 Emergency Department, Copenhagen University Hospital, Nordsjælland, Dyrehavevej 29, 3400 Hillerød, Denmark; 2 Department of Pulmonary & Infectious Diseases, Copenhagen University Hospital, Nordsjælland, Dyrehavevej 29, 3400 Hillerød, Denmark; 3 Department of Palliative Medicine, Copenhagen University Hospital, Herlev and Gentofte Hospital, Herlev Ringvej 75, 2730 Herlev, Denmark; 4 Neuroscience Centre, Copenhagen University Hospital, Rigshospitalet, Section 2091, Inge Lehmanns Vej 7, 2100 Copenhagen Ø, Denmark

**Keywords:** Interpretive description, Dualism, Non-malignant palliative care, Multidisciplinarity, Organizational changes, Resource utilization, Qualitative research

## Abstract

**Background:**

To improve the care of patients with advanced COPD and be able to address their palliative needs a new outpatient organization (CAPTAIN) was developed and implemented. CAPTAIN was inspired by best practice and existing guidelines and changed the traditional organization of an outpatient structure including the roles of nurses and doctors. Only sparse knowledge exists of the health professionals’ expectations and experiences to organizational changes in an outpatient setting. This insight is necessary as health professionals are key stakeholders in implementing new structures and successfully transforming knowledge into practice. The aim of this study was to explore the health professionals’ expectations and experiences of a new palliative out-patients structure for patients with advanced COPD.

**Methods:**

The design was interpretive description as described by Thorne. Focus groups and individual interviews were conducted with pulmonary nurses, pulmonary doctors and municipality nurses from 2014 to 2016.

**Results:**

The overall theme was dualism. Both nurses and doctors were pending between aspiration and concern in their expectations to the new structure, meanwhile their actual experiences were pending between perceived gain and improvements versus consequences with the new structure. Nurses’ and doctors’ existing practice was altered and the new structure required new ways for them to cooperate and ways in which skills from each profession were most efficiently utilized.

**Conclusion:**

**N**urses and doctors considered the new structure as a quality boost and it fulfilled their hope of improving the quality of care offered to patients with advanced COPD, however with increased work-related stress as a derived consequence.

## Background

Chronic Obstructive Pulmonary Disease (COPD) is a progressive and incurable disease with a global prevalence of more than 6 million people and COPD represents 5% of all deaths worldwide [1]. In Denmark the prevalence of COPD is estimated to 320.000 people, whereas 50.000 of these are categorized as having advanced COPD [2]. When the patients have reached the stage of advanced COPD, they have a high symptom burden, low functional capacity, frequent hospital admissions, and impaired quality of life. The need of palliative care for patients with advanced COPD is comparable with the needs of patients with advanced pulmonary cancer [3]. However, in Denmark more than 96% of patients referred to hospices or palliative teams are patients with cancer [4], and as a result patients with advanced COPD live their last year of life with unmet palliative needs and low quality of life [5].

COPD is characterized by an unpredictable illness trajectory with numerous exacerbations, each potentially life threatening. During the last past years there has been an increasing focus on the palliative needs of patients with COPD [6] and key guidelines recommend that patients with advanced COPD are offered an early integrated patient-centred care [6–9]. However, how an early patient-centred and integrated palliative care is organized and operationalized is unknown and further knowledge is warranted [4, 10–12].

By tradition in Denmark, the outpatient treatment of patients with COPD has been organized as an annual routine check by a pulmonary specialist accompanied by a pulmonary nurse. As outpatient visits were planned far in advance the visits had no link to the patients’ present needs or the progression of the disease. In case of acute exacerbations or symptom progression the patients were referred to their general practitioner (GP), municipal nurse, or an acute hospital admission. This traditional way of organizing an outpatient clinic failed to meet the individual and complex needs of patients with COPD and as a consequence the Department of Pulmonary Diseases at Nordsjællands Hospital designed and implemented a new palliative outpatient structure.

The Department of Pulmonary Diseases had a catchment area of 310.000 citizens in the Capital Region of Denmark, with a total of 650 patients affiliated the outpatient clinic. In average 240 patients died every year and 240 new patients were referred to the outpatient clinic. The new structure ‘Comprehensive And Prospective Treatment And Individual Nursing’ (CAPTAIN) was developed and implemented with a run-in-period of 2 years, and approximately 900 patients were enrolled in CAPTAIN during this period. The organisation is described in detail elsewhere [13]. The cornerstones of the new CAPTAIN structure was i) a multidisciplinary team-based approach, ii) nurses taking the responsibility of being case managers and iii) advanced care planning (ACP) [14]. The CAPTAIN-structure had an increased patient-centered approach compared to the current and traditional organisation [15]. The CAPTAIN structure was developed in collaboration between departmental management, a doctor and a nurse from the traditional pulmonary outpatient setting. The new structure was inspired by at that time limited existing knowledge and recommendations about COPD and palliation [4, 14, 16, 17].

The new structure required that both doctors and nurses changed their existing practices. The CAPTAIN-nurse had to take on the responsibility for establishing and maintaining an individualized patient and informal caregiver contact. In addition, the new structure required that both nurses and doctors altered their existing practice and found new ways to cooperate and ways to use skills from each profession in the best and most efficiently way.

To the best of our knowledge this way of organising a patient-centred outpatient structure for patients with advanced COPD is new, thus an unexplored research area with unknown perspectives and reactions of the involved health professionals. Nurses and doctors in the pulmonary outpatient clinic were introduced to the new structure by the chief physician and head nurse approximately 6 months before planned kick-off as their involvement was of paramount importance.

Several studies revealed that health professionals experienced barriers in providing palliative care for people with COPD [17–22]. Health professionals are key stakeholders in implementing new structures, and responsible of success of translating evidence into practice [23]. Insight in the involved health professionals’ perspective can provide awareness for leaders and other stakeholders and derived possibility to address barriers and enhance facilitators in the process of planning and implementation of new structures.

This study is the first of two, and will be followed by a study with the aim of exploring the patients’ perspective on the CAPTAIN-structure.

## Methods

### Aim

This study aimed to explore the health professionals’ expectations and experiences of a new palliative out-patients structure for patients with advanced COPD.

### Design

The methodology was interpretive description (ID) as described by Thorne [24]. ID is an inductive approach that focuses on meaning and how generated knowledge apply to clinical practice.

### Setting and participants

**Fig. 1:**
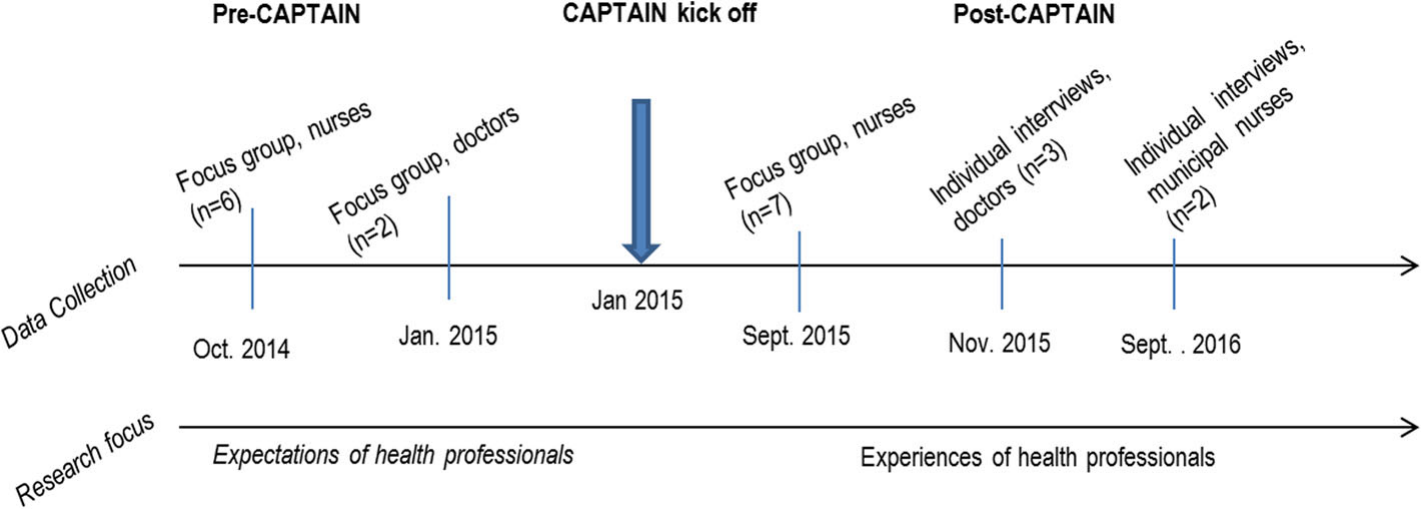
Data collection phase and related perspectives over time

We conducted a purposive sampling with a strategic identification based on the experiences and perspectives of key stakeholders in CAPTAIN. We defined the stakeholders of the CAPTAIN as pulmonary nurses, pulmonary doctors and primary nurses. In the following section the inclusion criteria for each stakeholder group are described. The concepts of pre and post CAPTAIN refer to the planning phase before implementing the new structure (pre) and the period after the CAPTAIN was introduced (post) (Fig. 1).

#### Pulmonary nurses

All nurses employed at the Department of Pulmonary Disease, Nordsjællands Hospital and affiliated to CAPTAIN (*n* = 6) participated in a focus group pre-CAPTAIN kick off and again 1 year after (*n* = 7). All the CAPTAIN nurses had a minimum of 5 years’ experience caring for patients with COPD and were considered as specialist pulmonary nurses. Four nurses from the focus group pre-CAPTAIN also participated in the focus group post-CAPTAIN. All nurses were female.

#### Pulmonary doctors

All three doctors employed at the Department of Pulmonary Disease, Nordsjællands Hospital and affiliated to CAPTAIN were invited to a focus group pre-CAPTAIN (*n* = 2) and to semi-structured interviews (*n* = 3) post-CAPTAIN. All doctors had more than 10 years’ experience as pulmonary consultants. One doctor was female.

#### Municipal nurses

Primary nurses from municipalities with patients affiliated to CAPTAIN participated in semi-structured interviews (*n* = 2). The municipalities were geographically located in the vicinity of North Zealand and the nurses had experience caring for patients with COPD affiliated CAPTAIN. The two municipal nurses were clinical specialists with several years of experience and dedicated caring for patients with COPD in their home environment. At the present time, the two municipal nurses represented at 100% of municipal nurses dedicated and highly experienced with caring for patients with COPD. All municipal nurses were female.

### Data collection

Focus group and semi-structured interviews were used to generate data. Nurses and doctors were all familiar with each other, and we found that focus groups were an effective and suitable strategy to collect multiple perspectives and diverse experiences. Our aim with the focus groups was to facilitate a synergetic discussion among the informants about expectations and experiences with CAPTAIN. If we had knowledge about a small number of informants, individual interviews were chosen over focus groups. Individual interviews gave us the opportunity to ask more in-depth questions and clarify insights generated through the focus groups. The data was collected from 2014 to 2016 (Fig. 1).

Data collection was conducted by two nurse-researchers with experiences in mediating focus groups and conducting interviews. Immediately after the focus groups or individual interviews the interviewer wrote a memo, describing the immediate experience of the interview and the initial analysis. By choice the interviewer was uninvolved in CAPTAIN and unfamiliar with the study participants prior to the focus group or individual interviews. Data collection and data analysis was performed simultaneously as an iterative process where the preliminary analysis gave direction to further data collection. The opening question for all interviews was: “*Please tell me about your expectations or experience with CAPTAIN”.*

### Data analysis

All interviews were transcribed verbatim by a medical secretary or the interviewer and lasted in average for focus group and semi-structured interviews, respectively 94 min (range 90–97 min) and 38 min (range 32–42). All transcripts were organized in QSR NVivo 11 © software (QSR International Pty ltd, Cardigan, UK).

In the first step of the analysis all transcriptions were read and reread in an attempt to immerse and become familiar with data. In the next step we started sorting and organizing data into broad-based and generic codes. First each focus group or interview was considered to be one unit of analysis. Later in the process data was divided into pre and post CAPTAIN, and the units of analysis was all data regardless of profession (nurse, municipal nurse or doctor) or data collection method (interview or focus group). Next, we searched for intuitive meaning units in the participants’ statements and the first themes and subthemes were identified. The themes and subthemes were explored by describing and discussing the content and meaning of each theme. In the next and final phase of the analysis we constructed multiple iterations of the themes by adding, re-organizing and merging several themes. As a final step in the analysis the first author (DB) read the original transcripts and the notes to ensure consistency among the themes and the informants’ descriptions.

### Rigour

To enhance credibility all steps in the research processes from the design of the study to the discussions of the themes have been conducted in discussion between two or more of the co-authors in accordance with the methodology ID [24]. All authors of this study are highly experienced in the field of COPD which has qualified the research process, the questions asked, the analysis and subsequent interpretation of the findings. This researcher triangulating has presumable reduced the risk of preconception and prejudices affecting the data collection and findings, and has contributed to keep focus on relevance of the findings for the clinical practice. The use of NVivo 11 was a valuable tool in strengthening dependability and the software contributed to transparency in the analysis process. Quotes underpinning the themes are presented in a concentrated and edited form to increase readability.

## Results

**Fig. 2:**
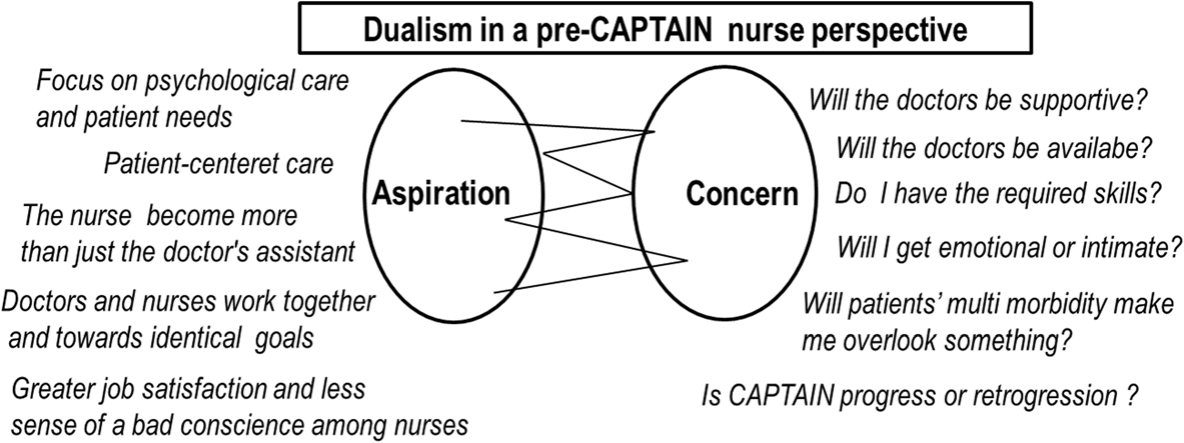
Theme and subthemes supported by categories originated from nurses’ perspectives pre-CAPTAIN

**Fig. 3:**
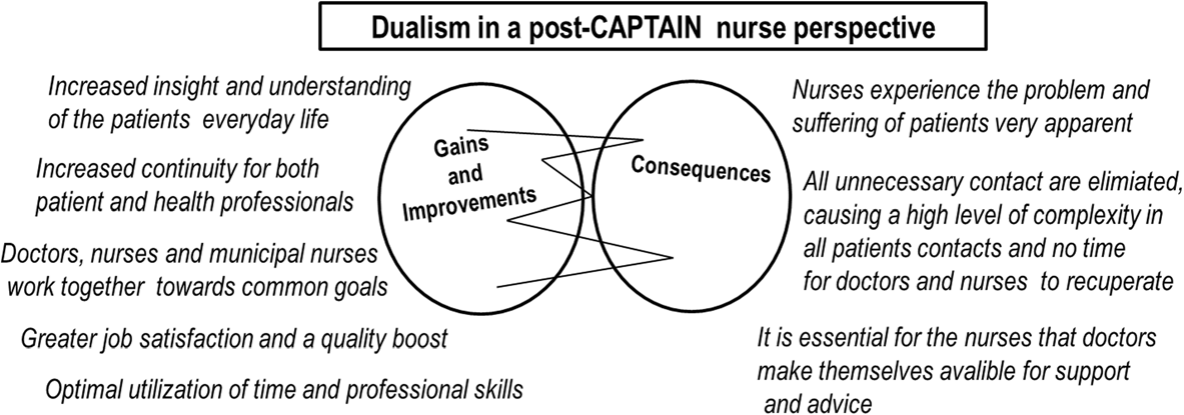
Theme and subthemes supported by categories originated from nurses’ perspectives post-CAPTAIN

The overall theme describing both the expectations and experience pre- and post-CAPTAIN was dualism, with the subtheme aspiration versus concern pre-CAPTAIN, and gain & improvement versus consequences post-CAPTAIN. The theme, subthemes and some of the underlying categories are illustrated in Figs. 2 and 3. The two figures are not an exhaustive illustration of the overall themes of the analysis, but merely an illustration of the nurses’ perspective.

### Aspiration and concern in the pre-CAPTAIN phase

Both nurses and doctors were characterized by dualism and pending between aspiration and concern in relation to what they expected the organizational changes would have of impact on both themselves and the patients.

*I am overwhelmed by the thought that everything will be broken down and changed, and that we as nurses must stand for it all. It is both a cool initiative but also overwhelming if suddenly it is expected that you could handle and know everything about it all. It was my first thought that one would have to know it all (Nurse, pre-CAPTAIN)*

Because the focus of the interviews was on expectations to the future it required that the informants were able to create abstractions. In response the nurses spoke of uncertainties of the new structure and listed several new questions they were preoccupied with (Fig. 2).

The doctors’ main concern was if they possessed the necessary communication skills to facilitate an advanced care planning (ACP) dialogue with the patient and their informal caregivers.

*I think it is important that we quickly establish the training of both doctors and nurses in the process of conducting ACP dialogues. What does the ACP contain, what is our role as doctors and how should we prepare to facilitate these dialogues? (pre-CAPTAIN, doctor)*

The doctors hoped that the new organizational structure would result in better utilization of their knowledge and skills and increase and strengthen the teamwork between doctors and nurses. By excusing doctors from routine visits of patients in a stable phase of COPD, they hoped to be able to focus on the complex cases and overall increase the quality of palliative care to patients with advanced COPD.

*Specifically, I see that we (doctors) see patients less often to routine visits than before and we give nurses more responsibility to establish and maintain contact with the patients. However, we also have more shared responsibility, doctors and nurses, in terms of communication. We have a shared responsibility to communicate and collaborate successfully, because if we do not, this project will not succeed. This project requires a great deal of teamwork and communication between all involved, including the general practitioners (GP) (pre-CAPTAIN, doctor).*

The nurses’ main concern was whether they possessed the adequate competences. A few nurses described a concern as to whether they were able to maintain professionalism in their interaction with the patients when they became better acquainted. They worried if they could deal with the intimate nature of the contact with severe ill patients and their relatives, and especially if they could keep patients out of their private sphere. Another concern was related to the teamwork with doctors. Nurses felt that multidisciplinary collaboration was essential for them in order to take on the increased responsibility and fulfill the role as a CAPTAIN-nurse. Nurses expressed it was imperative that doctors supported them and made themselves available for coaching as well as counseling.

### Gains and improvements and derived consequences in the post-CAPTAIN phase

The experiences of the health professionals are continuously characterized by a dualism, now pending between seeing the new structure as a quality boost and a positive change for patients but with the derived consequences triggering new concerns, predominantly among nurses. The new structure fulfilled aspirations and wishes described pre-captain by the health professionals. Although, the described concerns pre-CAPTAIN did not surface some of the concerns persisted in a vague or moderated form post-CAPTAIN (Fig. 3).

#### Gains and improvements but also concerns about derived consequences

Both nurses and doctors describe that the new CAPTAIN structure provide continuity and a better understanding of the patient’s everyday life and perceived values and preferences. However, this increased insight in the patients’ life and the awareness of suffering causes nurses to worry. Nurses realize that time does not permit them to render all the support patients are seeking or addressing all the problems patients are articulating.

*Patients can be clear about end of life wishes or not, some patients want to talk about death and others prefer not to. It's not hard to talk to patients about death, what's hard is the suffering. The suffering in their life; now and here. I cannot take the suffering away, but it's hard to witness and not being able to accommodate the patient's wish for me to do something to relieve their suffering, just something. It is the frustration of the chronic progressive disease, and we meet it all along. Sometimes the patients are at the bottom of the pit, where their life is so full of suffering, it can be difficult to witness. I wish I could pull a rabbit out of the hat and fix it (post-CAPTAIN, nurse).*

Nurses described how they together with the patient are forced to prioritize the current and the most important problem and decide what issues can be postponed to the next visit. This prioritizing highlights concern of quality and fear of overlooking anything important. The doctors, on the other hand, did not express this concern, neither on behalf of the nurses nor on their own behalf.

Increased insight and understanding of the patient struggles in everyday life was the result of caring for the same patient more continuously. More insight to the complexity of the patients’ problems combined with the available time and resources made nurses realize that to provide the best care nurses had to strengthen patients’ self-management capacity.

#### The CAPTAIN-structure redefines the role as a pulmonary doctor and nurse

Doctors described a change in their role as doctor. The new role was moving from being highly specialized toward a general pulmonologist more similar to a GP, as one doctor put it. Due to patient’s increased influence on the agenda in the meeting with the health professionals, the existing and traditional doctor/nurse/patient relationship was changed toward a more patient-centered and holistic approach. The nurses described how patients with COPD did not distinguish between pulmonary, cardiological, social, or psychological problems. Patients had problems that overall affected their life with COPD and therefore they seeked help to solve or alleviate the problem. Opposed to the health professionals patients did not consider organizational structures or divisions in anatomy and specialties. As a consequence the health professionals could not ignore or avoid “non-pulmonary medicine” issues.

#### The CAPTAIN-structure support cross-sectoral collaboration

Both nurses and doctors described that success in supporting the patients’ self-management capacity required an increased cross-sectoral collaboration, where the GP, municipal nurses and home care services were essential partners. This point of views was echoed by the municipal nurses. Municipal nurses described how CAPTAIN positively influence the quality of care delivered in the home due to the accessibility of the CAPTAIN-nurses, who provided them with an increased opportunity for seeking information about overall plans, treatment strategies, advice or support. The accessibility improved continuity for both patients and municipal nurses, and provided synergy as all health professionals across sectors were working towards identical goals.

*There is no doubt that the patient is very pleased to find that we work together [hospital and home care]. My experience is that we are a group working together in a kind of team setting and that one hand knows what the other is doing. I think that because we can communicate about everything patients get an increased feeling of safety (post-CAPTAIN municipality nurse).*

The municipality nurses described feeling convinced that the CAPTAIN-structure prevented unnecessary hospital admissions.

*I think CAPTAIN give us the opportunity to be more pro-active and preventive and thereby avoid the condition deteriorates and patient’s situation getting worse (post-CAPTAIN, municipality nurse).*

#### Enhancement of utilization of professional skills of each profession

Nurses, municipal nurses and doctors described how their competences and time was better used post-CAPTAIN compared to pre- CAPTAIN. However, this optimization of resources also had disadvantages. Especially doctors described how all contact with patients had a more clear purpose being either exacerbations or ACP. All unnecessary consultations were eliminated, resulting in high complexity in every patient contact. This high level of complexity was perceived by both nurses and doctors as demanding and at times mentally exhausting. Pre-CAPTAIN, the shift between complex and routine consultations gave the health professionals an opportunity “to breathe” and recuperate.

*Talking about death ... It's not a problem for me to be the patient’s companion on the road to death. I'm not crying because somebody dies, but I think afterwards I might need to take a breather. I really think I may need to have a break and find myself again (post-CAPTAIN, nurse).*

#### Multidisciplinarity was key

The ACP-dialogues was perceived as cornerstone of CAPTAIN by the doctors, whereas the nurses described the continuity and increased accessibility for both nurses and other collaborator as most important. The ACP was perceived as somewhat unpredictable as it could be very diverse and take different directions according to the agenda of the patients and their relatives. ACP was described by both doctors and nurses as much more than a dialogue about types and intensity of treatment. Doctors played a leading role facilitating the ACP-dialogue, although they did not consider themselves experts. The doctors described how they practiced and slowly improved over time. Both doctors and nurses considered the multidisciplinary teamwork as a strength in ACP, and highlighted that quality in ACP demanded continuous supervision to develop and maintain necessary skills.

*What patients express immediately after an ACP is that it was the best dialogue that have had with their doctor for years because the focus was not on lung function. Focus was on their anxiety and fear of being resuscitated by an emergency hospitalization (post-CAPTAIN nurse).*

Before CAPTAIN, nurses spoke of concerns about support and accessibility to the doctors, however this was un called for. Post CAPTAIN, nurses expressed seeing cooperation with doctors prerequisite for being able to fulfill the role as CAPTAIN-nurses. Doctors gave advice, guidance and support which still was considered essential for nurses in order to resume responsibility for establishing and maintaining contact with the patients throughout the disease trajectory. Being part of a multidisciplinary team and experiencing the support of doctors was described by nurses as of great importance to job satisfaction and provided motivation to take even more responsibility on. The nurses’ concerns pre-CAPTAIN about making mistakes or lacking the required skills were persistent post-CAPTAIN, although none of the nurses spoke of specific experiences to this point.

*I think it [the new role] is a huge challenge, it is great that we work very independently, but also a huge challenge because it is lurking at all time; have I overlooked something. (post-CAPTAIN, nurse)*

The doctors did not at any point substantiate this concern; in contrast they stated that nurses were considered highly competent and fully capable of solving the problems and ready to manage the increased responsibility.

## Discussion

To the best of our knowledge this study is the first to offer insight into the health professionals’ perspective on a new palliative outpatient structure aimed at improving treatment and care for patients with advanced COPD. This study does not explore the perceived value in a patient perspective, but exclusively focuses on the perspective of the health professionals, although it can be difficult to separate the two perspectives as they interact.

Our findings revealed that the health professionals’ expectations to the new CAPTAIN-structure were both hope and concern, and their actual experience were achieved gains versus derived consequences. It is well known that changes causes concern and an attitudinal reaction to changes can be driven by feelings of uncertainty, loss of control and fear of failure engendered by the changing event [23]. Although that all informants in our study were highly experience and we assumed that they had every reason to be confident about own skills, they still expressed fear of failure, albeit most pronounced among nurses. It was notable how the nurses repeatedly articulated concerns about their own skills. The doctors did not question own skills and expressed a great confidence in the nurses’ skills. The nurses were unaware of the doctors’ confidence in them and the doctors’ dedication to the multidisciplinary team and their intention to support them and make CAPTAIN a success. In general, it is considered unrealistic to eliminate all uncertainty in an initial phase of changing an existing structure; however it can be assumed that it would be beneficial to match expectations among nurses and doctors including discussion of present concerns in multidisciplinary meetings and supervision sessions.

A difference were also found in the description of what was required for respectively nurses and doctors to fulfill the role as CAPTAIN nurses or doctor. The doctors expressed a clear defined need for education and training in how to facilitate ACP dialogues, whereas the majority of nurses articulated a broader need for support and supervision related to complex cases and situations. This knowledge could be useful planning training- and education programs prior implementation of future organizational changes. Our findings indicate that it could be beneficial to combine formal education and training with sessions of supervision and scheduled time for interdisciplinary support.

According to Meleis’ theory of transition [25, 26], transition always includes a form of change and take place over time. The process of transition is diverse, complex, multi-dimensional and do always have a direction (purpose). It can be facilitated or inhibited by factors like perceived meaning, realistic expectations level of knowledge/skill, environment, level of planning, emotional and physical well-being. In our study, the health professionals described experiences that can be interpreted as both inhibitors and facilitators. Facilitators of the transition could have been supported by focusing and clarifying the meaning with the new structure, expectations to the health professionals including expectations and frame for the interdisciplinary collaboration. By doing so we might have reduced the level of concern, especially among nurses.

After the implementation of CAPTAN, the experience was that the new structure was a quality boost, but not without derived consequences. Especially the nurses described how they experienced a transition from being the doctor’s assistant to now having their own practice. Although, several nurses described a concern about becoming too emotional involved in the patients, none of them described episodes or experiences where this had actually happened or had caused a problem for them. However, the new structure exposed nurses to the patients suffering in a much greater extent than the traditional organization. Witnessing this suffering was described by nurses as hard and sometimes frustrating, but at the same time also seen as a consequence of the increased insight and understanding of the patient’s life which was perceived as something positive. The nurses’ fear of becoming too emotionally involved in the patient’s life were associated with the new role as a CAPTAIN-nurse. To be a CAPTAIN-nurse required more from the nurses than just objective measurements like lung function and inhalation techniques. In CAPTAIN there were a focus on forming human relationship beyond the pulmonary disease and that required a broader professionalism from the nurses. The doctors did not have these considerations which can be explained with the fact the roles of the doctors did not changes as fundamentally as the nurses.

Another important finding was that although all health professionals experienced a better utilization of their professional resources and competences due to elimination of uncomplicated patient contact it also had a negative impact on their ability to restitute and prepare for the next patient. Both doctor and nurses described episodes of being mentally extinguished and the experience of having an increased level of stress. Exposure to suffering and caring for complex patients and families are recognized stressors associated with increased risk of work-related stress [27]. It is therefore highly recommended that leaders and other stakeholders consider how to give the health professionals the opportunity to restitute and self-care and thereby lowering their level of stress. In our opinion it makes no sense or is feasible to implement a new structure that on one hand improve patientcare but on the other hand increases the health professionals’ level of stress and their derived risk of becoming ill.

This study is exploring the health professionals’ perspectives and will be followed by another qualitative study with the aim of exploring the patients’ perspectives. However, further research is needed to investigate the effectiveness of interventions like CAPTAIN and generate quantitative data about the impact on patients’ quality of life, hospitals admissions, length of stay and mortality. Future studies should also examine how cross-sectoral cooperation is best organized without demanding extra resources both from an organizational and a patient perspective, besides evaluating the impact of the altered organization on the health professionals’ workload and stress level.

As this paper very clearly illustrates, changing clinical practice is difficult and associated with an increased risk of stress among the health professionals, even though changes were well prepared in a group of experienced and collaborating nurses and physicians. At present, CAPTAIN is implemented as standard care for patients with severe COPD affiliated the pulmonary outpatient clinic. However, we do not consider the CAPTAIN structure as static, but rather it is under continuously development with the objective to reduce the perceived level of stress among the health professionals without lowering the quality for COPD patients or changing the fundamental framework of the CAPTAIN concept.

### Limitations

The health professional perspective was only explored through nurse or doctor perspective, and it could have contributed further knowledge if the mixed professional perspective had been explored. The nurses’ perspective was only explored through focus groups, and although focus groups contributes to multiple and diverse perspectives, the method do not allow for a deeper understanding of the participants experiences. It had to be noted the doctor focus group only consisted of two participants (*n* = 2), and that the municipality nurses’ perspective only was explored post-CAPTAIN.

All nurses were female, and two of three doctors male, raising the question if gender had influence on the prevalence of experienced concerns. The nurses in our study articulate in general more concerns than doctors and especially regarding their level of both personal and professional competences. The literature illustrates, although somewhat inconsistent, that women respond with higher level of stress to work-related stressors, which implementation of a new structure is considered to be [28].

## Conclusions

This study illustrates how it is possible, although challenging, to change the structure and roles of nurses and doctors in a pulmonary outpatient clinic for patients with advanced COPD. Both doctors and nurses experienced the new structure as a quality boost and it fulfilled the hope of improving the quality of care offered to patients with advanced COPD. The insight gained through this study highlight the need for addressing concerns continuously during both the planning and implementation phase, and in a multidisciplinary forum.

Especially, the nurses were insecure about possessing the required skills and wandered if the doctors would support them to the extent they needed.

Both doctors and nurses expressed that the new structure optimized the utilization of their professional resources, although with the consequence that all uncomplicated patient-contacts were eliminated. This meant that there was no opportunity to restitute between patient contacts, which increased the experience of work-related stress and in some cases led to feeling of being mentally exhausted.

Our findings may guide leaders and other stakeholders considering or standing before changing an existing organizational outpatient-structure to improve or implement palliative care to patients with COPD. We recommend that both leaders, nurses and doctors are aware and discuss pros and cons prior to implementing a new palliative structure. In a structure like CAPTAIN, the increased awareness of the patients’ high symptom burden and suffering means that far more patients will be recognized as complex. The multidisciplinary team must have the time required to address this complexity, otherwise there is an increased risk of stress and dissatisfaction within the multidisciplinary team. However, we find it possible that by planning ongoing supervision and multidisciplinary dialogues this stress can be prevented or reduced. Although, this study is conducted in the context of a pulmonary outpatient clinic, our findings may be transferrable to other in-hospital settings and groups of patients with non-malign diseases with less tradition for palliative care as dementia and heart failure. This assumption is based on the health professionals’ experience that the support the patients demanded was about management of life with a severe disease rather on specific pulmonary issues.

## Abbreviations

ACP: Advanced Care Planning
CAPTAIN: Comprehensive and Prospective Treatment and Individual Nursing
COPD: Chronic Obstructive Pulmonary Disease
GP: General Practioner
ID: Interpretive Description
